# Comparison of plasma and salivary HIV loads determined via a coupling of the Abbott HIV detection system with the DNA Genotek OMNIgene™ DISCOVER (OM-505) kits

**DOI:** 10.1186/1471-2334-14-S3-P80

**Published:** 2014-05-27

**Authors:** David J Speicher, S Saravanan, N Kumarasamy, K Rangananthan, NW Johnson

**Affiliations:** 1 Molecular Basis of Disease Research Program, Griffith Health Institute, Griffith University, Queensland, Australia; 2 Population & Social Health Research Program, Griffith Health Institute, Griffith University, Queensland, Australia; 3 YR Gaitonde Centre for AIDS Research and Education, Chennai, India; 4 Ragas Dental College, Chennai, India

## Background

The DNA Genotek OMNIgene™ DISCOVER (OM-505) kits are designed to collect and store saliva at room temperature before the extraction and detection of DNA and RNA. Utilizing the OM-505 we determined the HIV salivary viral loads (SVL), which were compared with plasma viral loads (PVL).

## Methods

SVL and PVL were determined on 40 HIV-positive ART naïve patients presenting at YRG CARE. Saliva was collected with the OM-505, incubated at 50°C for 1 hour. Prior to extraction 70 mL isopropanol was mixed with 800mL OM-505. From OM-505 and plasma, RNA was extracted automatically on the Abbott m2000sp. HIV loads were determined with the Abbott m2000rt system.

## Results

A calibration curve produced by 10-fold dilutions of HIV virion in HIV negative saliva collected in the OM-505 was linear (*R*^2^=0.9951) from 57,273 to 621 HIV copies/mL. In clinical isolates, PVL averaged 431,865 HIV copies/mL (range: 62 to 7,604,620 HIV copies/mL) whilst SVL averaged 23,267 HIV copies/mL (range: 153 to 220,104 HIV copies/mL). SVL was not detected in 15 samples and could not be determined in 5 samples due to viscosity and cellular debris causing problems during extraction. In 12/17 patients SVL was lower than PVL.

## Conclusion

HIV, if present, can be detected accurately in saliva down to 621 HIV copies/mL. SVL does not correlate with PVL and thus cannot be used to accurately determine HIV carriage, but in most cases HIV shedding is low or nonexistent.

